# Society Meetings

**Published:** 1899-08

**Authors:** 


### SOCIETY MEETINGS.

The American Association of Obstetricians and Gynecologists will hold its twelfth annual meeting in the Assembly room of the Denison House, Indianapolis, Ind., Tuesday, Wednesday and Thursday, September 19, 20 and 21, 1899.

PAPERS PROMISED.

1. The president's address, Edward J. Ill, Newark.

2. Three rare cases of kidney cyst, J. F. Baldwin, Columbus.

3. Postpartem repair of lacerations of the cervix uteri, Clinton Cushing, Washington.

4. The gonorrheal puerperium, Charles G. Cumston, Boston.

5. ..................., Rufus B. Hall, Cincinnati.

6. Injury to ureter in abdominal section, L. H. Dunning, Indianapolis.

7. ..................., J. B. Murphy, Chicago.

8. Coccygeal dermoid fistulæ, Robert T. Morris, New York.

9. ..................., X. O. Werder, Pittsburg.

10. ..................., Walter A. Jayne, Colorado.

11. ..................., C. C. Frederick, Buffalo.

12. ..................., Walter B. Dorsett, St. Louis.

13. ..................., J. Henry Carstens, Detroit.

14. Choice of method for total hysterectomy and some points of technique, B. Sherwood-Dunn, Boston.

15. Present position of gall-stone surgery with report of cases, William Wotkyns Seymour, Troy.

16. ..................., John B. Deaver, Philadelphia.

17. What shall we do with the post-operative hemorrhage of celiotomy? D. Tod Gilliam, Columbus.

18. ...................., M. Rosenwasser, Cleveland.

19. Choice of operative method from a mortality point of view, Joseph Price, Philadelphia.

20. Shall we operate during the viability of the fetus when at or near term? L. H. Dunning, Indianapolis.

21. The deleterious influence of tea and coffee in a certain class of gynecological cases, Walter B. Chase, Brooklyn.

22. One form of ovarian disease not generally recognised, W. H. Humiston, Cleveland.

23. Personal experience with uterine fibroids, Henry D. Ingraham, Buffalo.

24. Midsummer operations, Joseph Price, Philadelphia.

25. Observations respecting the symptoms and treatment of the menopause, Augustus P. Clarke, Cambridge.

26. Treatment of inertia and subinvolution of the uterus, Charles Stover, Amsterdam.

27. A simple, effective and esthetic operation for shortening the round ligaments, H. W. Longyear, Detroit.

28. Some observations, chiefly clinical, upon the temperature after intraperitoneal operations, L. S. McMurtry, Louisville.

29. Rupture of the puerperal uterus, with cases, James F. W. Ross, Toronto.

30. In memoriam—The life and character of Lawson Tait, by Charles A. L. Reed, of Cincinnati.

The titles of papers are announced in the order of their reception. The permanent program will be classified and issued August 25th, after which date no further titles can be added.

☞ It is considered especially desirable that each author of a paper forward to the secretary a concise argument thereof, under three or four separate heads, to be printed in the permanent program. This will add to the interest of the discussions.

All members of the medical profession are cordially invited to attend the scientific sessions.

---

The American Proctological Society is the name of a new national medical association organised at Columbus, Ohio, June 7, 1899. The society is formed for the study of the diseases of the rectum, and its membership is composed of the following prominent rectal specialists, who became charter members: Joseph M. Mathews, Louisville; Joseph B. Bacon, Chicago; Leon Straus, St. Louis; B. Merrill Ricketts, Cincinnati; Thomas Charles Martin, Cleveland;

S. G. Gant, Kansas City; J. Royal Pennington, Chicago; James P. Tuttle, New York City; Samuel T. Earle, Baltimore; Lewis J. Adler, Jr., Philadelphia; Charles C. Allison, Omaha; A. Bennett Cooke, Nashville; George J. Cook, Indianapolis; George B. Evans, Dayton, Ohio; William M. Beach, Pittsburg.

The following officers were elected: President, Joseph M. Mathews, Louisville; vice-president, James P. Tuttle, New York City; secretary-treasurer, William M. Beach, Pittsburg; board of councilors, Samuel T. Earle, Baltimore; A. Bennett Cooke, Nashville, J. Royal Pennington, Chicago.

The next meeting of the association will be held at Washington, D. C., in May, 1900.

---

THE American Electro-therapeutic Association will hold its ninth annual meeting at Washington, D. C., September 19, 20, 21, 1899. The president, Dr. F. B. Bishop, appointed the following committee of arrangements: D. Percy Hickling, chairman, Joseph Taber Johnson, G. Lloyd Magruder, Z. T. Sowers, Robert Reyburn, G. Betton Massey, Charles R. Luce, Elmer Sothoron, Llewellyn Eliot, Clifton Mayfield.

Willard's Hotel has been chosen for the headquarters and special rates have been made for all interested in this meeting. Many able papers have been promised and a very successful scientific meeting is assured. There will be a large and varied exhibition of electro-therapeutic apparatus in Willard's Hall during the meeting of the association.

---

THE Niagara Falls Academy of Medicine held its last regular meeting previous to adjournment for the summer vacation at the office of the president, Dr. W. A. Scott, Monday evening, July 10, 1899.

